# Ensuring availability of respiratory medicines in times of European drug shortages

**DOI:** 10.1183/13993003.01634-2024

**Published:** 2024-11-07

**Authors:** Job F.M. van Boven, Arzu Yorgancioglu, Nicolas Roche, Omar S. Usmani

**Affiliations:** 1 Department of Clinical Pharmacy and Pharmacology, Groningen Research Institute for Asthma and COPD (GRIAC), University Medical Center Groningen, University of Groningen, Groningen, The Netherlands; 2 Lung Alliance Netherlands, Amersfoort, The Netherlands; 3 Department of Pulmonology, Medical Faculty, Celal Bayar University, Manisa, Turkey; 4 Department of Respiratory Medicine, APHP Centre, Institut Cochin (UMR 1016), Assistance Publique-Hôpitaux de Paris, Cochin Hospital, University Paris Cité, Paris, France; 5 National Heart and Lung Institute, Imperial College London, London, UK

## Abstract

It is of utmost importance that medicines are available at all times for our patients. Historically, medication unavailability has typically, if not exclusively, affected low- and middle-income countries [1]. More recently however, drug shortages have also been reported in high-income European countries [2]. Drug shortages have negative health consequences for patients [3], and a profound economic impact, with the need to resort to more expensive alternatives and demands on healthcare professionals’ time to find, prescribe and dispense alternatives [4].

## Shareable abstract

**European policy should stimulate, not oppose, availability of respiratory medicines in times of drug shortages** https://bit.ly/4eBjxkb

*To the Editor*:

It is of utmost importance that medicines are available at all times for our patients. Historically, medication unavailability has typically, if not exclusively, affected low- and middle-income countries [1]. More recently however, drug shortages have also been reported in high-income European countries [2]. Drug shortages have negative health consequences for patients [3], and a profound economic impact, with the need to resort to more expensive alternatives and demands on healthcare professionals’ time to find, prescribe and dispense alternatives [4].

Shortages occur due to multiple reasons: manufacturing, transportation or importation issues, sudden demands (*e.g.* COVID-19), and political (*e.g.* Brexit) or commercial decisions [5]. In response, individual European countries have taken several actions, including requiring manufacturers and wholesalers to stock supply reserves, enforce export bans and ration drugs nationally, as well as financial incentives and sanctions [5]. However, increasing stock reserves may lead to higher expenses and also to environmental waste, especially if the drugs are not used before expiry. Therefore, maintaining a balance between availability, affordability and sustainability is important (figure 1).

**FIGURE 1:**
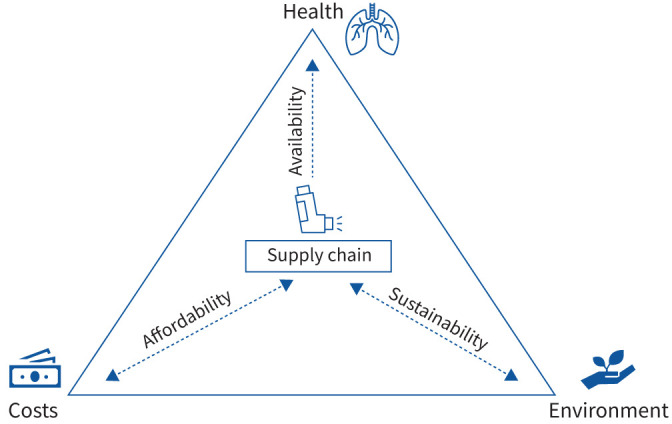
Navigating the tricky triangle of availability, affordability and sustainability of respiratory medicines.

In December 2023, the European Commission published the first Union Critical Medicines list, containing over 200 active pharmaceutical substances [6]. “Critical medicines” have been selected based on 1) therapeutic indication and 2) the availability of appropriate alternatives. For listed medicines, specific European Union (EU)-wide policy, such as promoting diversification of suppliers and maintaining production within the EU, should secure supplies.

Notably, only a few respiratory medicines are on the EU's list: salbutamol, acetylcysteine, ephedrine and ipratropium. Drugs for the treatment of tuberculosis are well covered. There are, however, some noteworthy omissions. For example, critical medicines for the treatment of cystic fibrosis (*e.g.* pancreatic exocrine replacement therapies) are completely lacking [6]. Furthermore, the list does not contain inhaled corticosteroids, the cornerstone of airway disease management, while they are included in the World Health Oragnization's Essential Medicines list. Importantly, the inhaler type of the two listed inhaled medicines, salbutamol and ipratropium, is not specified, which is critical as dry powder inhalers, in contrast to pressurised metered dose inhalers (pMDIs), are unsuitable for young children, the elderly and those with insufficient inhalation capacity during acute lung attacks.

While the EU's list aims to stimulate drug availability by diversification of suppliers, active country-level health economic policy, although well-meant, may adversely affect this diversification. For example, in the Netherlands, a generic drug pricing policy was introduced in 2005 [7], where only the generic drug with the lowest negotiated price was reimbursed, leading to the Netherlands having the lowest price for generics in Europe. However, indirectly, this led to heavy reliance on just one or two supply chains as other generic manufacturers held low stock or even revoked their registration in the Netherlands. Relying on a single supply chain may jeopardise drug availability [8] and compromise patients’ health while increasing healthcare costs through greater needs for acute care. Indeed, in Spring 2024, a Lung Alliance Netherlands survey (n=640) revealed that 4.5% of healthcare professionals reported severe patient complications, such as emergency department visits and hospital admissions, linked to salbutamol pMDI unavailability [9].

For inhaled respiratory medicines in particular, recent environmental policy could inadvertently restrict the diversification of drug supply chains, and thus increase the risk of their unavailability [10]. Policymakers have called for restrictions on the use of F-gases (fluorinated gases that have global warming potential (GWP)) and per- and poly-fluoroalkyl substances (PFAS; “forever chemicals” that are highly persistent environmental substances) as propellants in pMDIs [11]. Current pMDIs contain propellants HFA-134a or HFA-227 (F-gases with ∼1500–3000 higher GWP than carbon dioxide). In response to the F-gas regulation, the pharmaceutical industry has worked on two alternative propellants that have substantially lower GWP: HFA-152a and HFO-1234ze. However, HFA-152a still falls under the restrictive F-gas regulation, where HFO-1234ze, with near-zero GWP, may potentially fall under the newly proposed PFAS restriction. Both the F-gas and PFAS regulation may thus reduce the availability of pMDIs. Of further consideration, the European plastics strategy, which includes regulations regarding recyclability and use of microplastics, may affect availability of all inhalers [12].

Overall, there is great concern that European regulations may lead to fewer respiratory medicines available, compromising the everyday treatment of our patients. In 2024, the respiratory community finds itself under intense pressure in the interests of its patients, navigating the tricky triangle of drug availability, affordability and sustainability (figure 1). Collective advocacy from the respiratory community to policymakers and governments in order to safeguard respiratory medicine availability is needed now more than ever. While researchers, industry, healthcare professionals and all involved stakeholders need to continuously aim at minimising the environmental impact of respiratory treatments, and affordability issues clearly need to be addressed, we call for a very careful consideration of policies in Europe, keeping respiratory medicine availability and patients’ respiratory health a top priority.

## Shareable PDF

10.1183/13993003.01634-2024.Shareable1 This one-page PDF can be shared freely online.
Shareable PDF ERJ-01634-2024.Shareable
