# Supplementary material for: Ensuring availability of respiratory medicines in times of European drug shortages
Source: Eur Respir J. 2024 Nov 7;64(5):2401634. doi: 10.1183/13993003.01634-2024 (PMC11540981; doi:10.1183/13993003.01634-2024)
Supplement: Supplementary file 1 [file ERJ-01634-2024.Shareable.pdf]

EUROPEAN RESPIRATORY JOURNAL
CORRESPONDENCE
J.F.M. VAN BOVEN ET AL.

SHAREABLE PDF

Ensuring availability of respiratory medicines in times of European drug shortages

Job F.M. van Boven [1,2], Arzu Yorgancioglu[3], Nicolas Roche [4] and Omar S. Usmani[5]

[1]Department of Clinical Pharmacy and Pharmacology, Groningen Research Institute for Asthma and COPD (GRIAC), University Medical Center Groningen, University of Groningen, Groningen, The Netherlands. [2]Lung Alliance Netherlands, Amersfoort, The Netherlands. [3]Department of Pulmonology, Medical Faculty, Celal Bayar University, Manisa, Turkey. [4]Department of Respiratory Medicine, APHP Centre, Institut Cochin (UMR 1016), Assistance Publique-Hôpitaux de Paris, Cochin Hospital, University Paris Cité, Paris, France. [5]National Heart and Lung Institute, Imperial College London, London, UK.

Corresponding author: Job F.M. van Boven (j.f.m.van.boven@rug.nl)

Check for updates

Shareable abstract (@ERSpublications)
European policy should stimulate, not oppose, availability of respiratory medicines in times of drug shortages https://bit.ly/4eBjxkb

Cite this article as: van Boven JFM, Yorgancioglu A, Roche N, *et al*. Ensuring availability of respiratory medicines in times of European drug shortages. *Eur Respir J* 2024; 64: 2401634 [DOI: 10.1183/13993003.01634-2024].

This extracted version can be shared freely online.

Copyright ©The authors 2024.

This version is distributed under the terms of the Creative Commons Attribution Licence 4.0.

Received: 19 Aug 2024
Accepted: 13 Sept 2024

To the Editor:

It is of utmost importance that medicines are available at all times for our patients. Historically, medication unavailability has typically, if not exclusively, affected low- and middle-income countries [1]. More recently however, drug shortages have also been reported in high-income European countries [2]. Drug shortages have negative health consequences for patients [3], and a profound economic impact, with the need to resort to more expensive alternatives and demands on healthcare professionals' time to find, prescribe and dispense alternatives [4].
